# Extraction, purification, characterization, and *in vitro* anticoagulant activity of polysaccharides from *Cordyceps militaris*

**DOI:** 10.3389/fnut.2026.1915697

**Published:** 2026-09-17

**Authors:** Cai-fang Li, Li-gan Lu, Jun-shang Liu, Cheng-qi Liu, Yan Ma, Shuang-shuang Zhang, Zi-heng Jin, Chang-yang Ma, Jin-mei Wang

**Affiliations:** 1 Department of Pharmacy, Xinxiang Central Hospital, Xinxiang, China; 2 Henan University Hospital, Henan University, Kaifeng, China; 3 National R&D Center for Edible Fungus Processing Technology, Henan University, Kaifeng, China; 4 College of Agriculture, Henan University, Kaifeng, China; 5 Henan Key University-Enterprise R&D Center for Special Foods, Kaifeng, China

**Keywords:** anticoagulant activity, compositional analysis, *Cordyceps militaris*, polysaccharides, structural characterization

## Abstract

**Objective:**

To elucidate the anticoagulant effects of *Cordyceps militaris* polysaccharides and provide a scientific basis for their application in functional foods and biomedicine.

**Methods:**

Crude polysaccharide (CC-1) was extracted by water extraction and ethanol precipitation, and then purified by papain-mediated deproteinization and dialysis to obtain polysaccharide CC-2. Chemical composition was analyzed using the phenol–sulfuric acid method, the meta-hydroxybiphenyl method, and a BCA protein assay kit. Molecular weight was determined by high-performance gel permeation chromatography (HPGPC). Monosaccharide composition was analyzed by PMP pre-column derivatization HPLC (PMP-HPLC). Structural features were characterized by Fourier transform infrared (FT-IR) and ultraviolet (UV) spectroscopy, and scanning electron microscopy (SEM). *In vitro* anticoagulant activity was evaluated by measuring activated partial thromboplastin time (APTT), prothrombin time (PT), thrombin time (TT), and fibrinogen (FIB) levels.

**Results:**

The yield of CC-2 was 2.16%. Compared with CC-1, CC-2 showed significantly higher total sugar and uronic acid contents and a decreased protein content. The molecular weight of CC-2 was 5908.612 Da. Its monosaccharide composition consisted primarily of rhamnose, glucuronic acid, glucose, galactose, and xylose in a molar ratio of 5:1:4:11:4. FT-IR indicated the presence of acetyl groups and a furanose ring conformation. SEM revealed a microstructure composed of irregular lumps and spherical particles. *In vitro*, CC-2 significantly prolonged PT, APTT, and TT (*P* < 0.001) by approximately 2.10, 2.46, and 2.79 fold, respectively, and significantly reduced FIB levels (*P* < 0.001). Its FIB-lowering effect was superior to that of the positive control, breviscapine.

**Conclusion:**

*Cordyceps militaris* polysaccharides exhibit significant *in vitro* anticoagulant activity, acting through potential multi-pathway mechanisms and pronounced fibrinogen reduction. These findings support their potential development as functional food ingredients or biomedical agents for the prevention of thrombotic cardiovascular and cerebrovascular diseases.

## 1 Introduction

Cardiovascular and cerebrovascular diseases (CVDs) represent a major global health threat. The situation is particularly alarming in China, where the number of patients with CVDs has exceeded 330 million according to The China Cardiovascular Health and Diseases Report 2024. Stroke, characterized by high incidence, disability, recurrence, and mortality rates, is a leading cause of death and long-term disability among Chinese adults, imposing a heavy burden on patients’ families and the healthcare system. Strokes are primarily classified as ischemic (accounting for approximately 85% of cases) and hemorrhagic. The pathological core of ischemic stroke involves thrombus formation, leading to cerebral vascular embolism (1).

Current antithrombotic therapies, including low-molecular-weight heparin, aspirin, and clopidogrel, can inhibit thrombus formation. However, their long-term clinical application is often limited by adverse effects, such as a high risk of bleeding, allergic reactions, gastric mucosal damage, and impaired liver function. Therefore, there is an urgent need to develop safe, efficacious, and low-side-effect anticoagulant agents from natural sources.

Edible mushrooms are renowned for their nutritional and medicinal properties. Over 6,000 species have been identified in nature, with more than 2,000 confirmed as edible (2). Polysaccharides, the primary bioactive components of edible fungi, have been extensively studied and demonstrated to play various health-regulatory roles, including modulation of the gut microbiota and exertion of hypoglycemic effects (3, 4).

Cordyceps is a complex genus of entomopathogenic fungi with a long history in traditional Chinese medicine (5). Modern research has confirmed that Cordyceps-related preparations can reduce blood viscosity and improve hemorheological characteristics. For instance, Cordyceps sinensispowder, its mycelium, and cordycepin have been shown to inhibit platelet activation and aggregation, demonstrating promising anticoagulant and antithrombotic potential (6, 7).

*Cordyceps militaris*, also known as Northern Cordyceps, is an edible mushroom belonging to the genus Cordyceps (8). In 2014, it was approved as a new food ingredient in China. This fungus is primarily distributed in the temperate and cold regions of Asia, Europe, and North America (9). Modern pharmacological studies indicate that *C. militaris* contains a diverse array of bioactive compounds, mainly polysaccharides and cordycepin, which exhibit immunomodulatory, sedative-hypnotic, antitumor, and antioxidant effects (10–14). However, to date, no studies have reported on the anticoagulant activity of polysaccharides derived from *C. militaris*.

Therefore, this study focuses on the isolation, purification, and physicochemical characterization of *C. militaris* polysaccharides, followed by an *in vitro* evaluation of their anticoagulant activity. The aim is to clarify their anticoagulant effects and provide experimental evidence and theoretical support for their potential application in functional foods and biomedicine.

## 2 Materials and methods

### 2.1 Materials and reagents

*Cordyceps militaris* fruiting bodies were purchased from the Xuchang Herbal Medicine Market (Henan, China) in August 2025.

Analytical grade reagents, including sulfuric acid, phenol, anhydrous ethanol, sodium chloride, pyridine, sodium tetraborate, and anhydrous sodium sulfate, were obtained from various domestic suppliers in China. Trifluoroacetic acid (TFA) was purchased from Merck & Co., Inc. (USA). Monosaccharide standards (D-glucose, L-rhamnose, L-arabinose, D-xylose, D-mannose, D-galactose, D-fructose, L-fucose, D-galacturonic acid, D-glucuronic acid, D-lactulose) were from Dr. Ehrenstorfer GmbH (Germany). 1-phenyl-3-methyl-5-pyrazolone (PMP) was from Sigma-Aldrich (USA). Purified water was from Hangzhou Wahaha Group Co., Ltd. (China). A BCA protein assay kit was used for protein quantification.

### 2.2 Instruments and equipment

The instruments used included a Model 85-2B Magnetic Stirrer, an N-1300 Rotary Evaporator, an LGJ-10FD Vacuum Freeze Dryer, a Multiskan Go Full-Wavelength Microplate Reader (Thermo Fisher Scientific, USA), a JW-2019H Medical Centrifuge, an ME104 Electronic Balance (Mettler-Toledo), a DHG-9053A Electric Forced-Air Oven, a UV-2600 UV-Vis Spectrophotometer (Shimadzu, Japan), a Nicolet iS50 FT-IR Spectrometer (Thermo Fisher Scientific, USA), an MV-100 Vortex Mixer, a DZTW Electronic Temperature-Controlled Heating Mantle, a JEOL JSM-7610F Plus Field-Emission Scanning Electron Microscope (JEOL Ltd., Japan), a nano ZS nanoparticle size analyzer (Malvern Instruments Ltd., UK), and a RAC-030 automated coagulation analyzer (Rayto Life and Analytical Sciences Co., Ltd., China).

### 2.3 Extraction and purification of *C. militaris* polysaccharides

*C. militaris* fruiting bodies (4.9 kg) were chopped and defatted by soaking in 75% ethanol (1:10, w/v) three times for 72 h each. The residue was air-dried and then extracted with distilled water (1:20, w/v) at 80 °C for 3 h. This extraction process was repeated three times. The combined filtrates were concentrated under reduced pressure, and the polysaccharides were precipitated by adjusting the solution to a final ethanol concentration of 75% and storing at 4 °C for 24 h. The precipitate was collected, air-dried, and designated as crude polysaccharide CC-1 (Yield: 9.85%). In preliminary experiments, both papain enzymatic hydrolysis and the Sevag method (chloroform/n-butanol, 4:1, v/v) were tested for protein removal. The two methods showed comparable deproteinization efficiency. The papain method was selected for subsequent experiments because it avoids the use of large volumes of organic solvents and reduces the risk of polysaccharide degradation. CC-1 was redissolved, and papain (20% of the mass of CC-1) was added for enzymatic hydrolysis at 60 °C for 1 h. After enzyme inactivation, the mixture was centrifuged at 8,000 rpm for 10 min to remove denatured proteins. The supernatant was then dialyzed against distilled water for 48 h using a dialysis bag with a molecular weight cutoff (MWCO) of 8000–14000 Da to remove salts and low-molecular-weight impurities. The retentate was freeze-dried to yield the *C. militaris* polysaccharide, named CC-2. The yield of CC-2 was 2.16% based on the initial dried material.

### 2.4 Chemical composition analysis

The neutral sugar content was determined by the phenol-sulfuric acid method using glucose as the standard (15). The uronic acid content was measured by the meta-hydroxybiphenyl method using glucuronic acid as the standard (16). The protein content was determined using a BCA protein assay kit following the manufacturer’s instructions.

### 2.5 Molecular weight determination

The Mw of the polysaccharide was determined by high-performance gel permeation chromatography (HPGPC). Dextran standards with different Mws (1–500 kDa) and the CC-2 sample (2 mg each) were dissolved in a 0.1 mol/L NaNO_3_ aqueous solution to a concentration of 2 mg/mL and filtered through a 0.22 μm membrane. The analysis was performed on a Shimadzu LC-20ADXR system equipped with a 20A differential refractive index detector and a Shodex OHpak SB-806 HQ column (10 μm, 8 mm × 300 mm). The mobile phase was 0.1 mol/L NaNO_3_ at a flow rate of 0.5 mL/min, with a column temperature of 35 °C and an injection volume of 40 μL. A standard curve was generated by plotting the logarithm of Mw (Log Mw) against retention time. The Mw of CC-2 was calculated based on its retention time using the regression equation.

### 2.6 Structural characterization

#### 2.6.1 Monosaccharide composition analysis

The monosaccharide composition was determined by a PMP pre-column derivatization HPLC method, with slight modifications (17). Briefly, 4 mg of polysaccharide was hydrolyzed with 1 mL of TFA (4 mol/L) at 110 °C for 4 h. After repeated co-evaporation with methanol to remove residual TFA, the hydrolysate was dissolved in distilled water. The monosaccharide standards and hydrolysate were derivatized with PMP, and the derivatives were analyzed by HPLC on an Agilent TC-C18 column (250 mm × 4.6 mm, 5 μm). The mobile phase consisted of acetonitrile and 0.1 mol/L phosphate buffer (pH 6.7) with gradient elution at a flow rate of 1 mL/min. Detection was performed at 254 nm, and monosaccharides were quantified using an internal standard method.

#### 2.6.2 UV and FT-IR spectroscopy analysis

A solution of CC-2 in distilled water was scanned with a UV-Vis spectrophotometer in the range of 200–400 nm.

For FT-IR analysis, dried polysaccharide powder was ground with spectroscopic-grade KBr, pressed into a pellet, and scanned over the 4000–500 cm^−1^ range.

#### 2.6.3 Particle size distribution analysis

The average particle size (Z-Ave) and polydispersity index (PdI) of polysaccharide solutions (0.1 mg/mL) were determined at 25 °C using a nanoparticle size analyzer. Each sample was measured in triplicate.

#### 2.6.4 Scanning electron microscopy (SEM)

A 1 mg/mL polysaccharide solution was prepared, rapidly frozen in liquid nitrogen, and freeze-dried. The dried sample was mounted on a conductive adhesive, sputter-coated with gold, and observed using a field-emission scanning electron microscope at an acceleration voltage of 5.0 kV.

### 2.7 *In vitro* anticoagulant assay

Blood was collected from the ear lobe of healthy rabbits into tubes containing 3.2% sodium citrate (9:1 v/v). Platelet-poor plasma was obtained by centrifugation at 3,500 rpm for 10 min at 4 °C. The *in vitro* anticoagulant activity was evaluated according to the method of Wang (18). CC-2 was dissolved in normal saline to a concentration of 5 mg/mL. Normal saline and an Breviscapine solution (20 mg/mL) served as the negative and positive controls, respectively. Breviscapine was selected as the positive control due to its clinical use in cardiovascular disease and its reported effects on plasma coagulation activity (18). Four coagulation parameters – APTT, PT, TT, and FIB – were measured using an automated coagulation analyzer.

### 2.8 Statistical analysis

All experiments were performed in triplicate, and results are expressed as the mean ± standard deviation (X ± SD). Data were analyzed using GraphPad Prism 10.0 software. Statistical comparisons were made using one-way analysis of variance (ANOVA). A value of *P* < 0.05 was considered statistically significant.

## 3 Results and discussion

### 3.1 Extraction yield and chemical composition

The crude polysaccharide CC-1 was obtained as a water-soluble extract. After deproteinization and dialysis, the yield of the polysaccharide CC-2 was 2.16%. As shown in Table 1, CC-2 exhibited a significantly higher total sugar content (52.49 ± 1.54%) and uronic acid content (2.37 ± 0.48%) compared to CC-1 (31.95 ± 0.48% and 1.74 ± 0.03%, respectively). Correspondingly, the protein content decreased from 24.83 ± 4.28% in CC-1 to 21.69 ± 5.52% in CC-2, demonstrating the effectiveness of the purification process.

**TABLE 1:** Neutral sugar, uronic acid, and protein content of *C. militaris* polysaccharides.

Sample	Neutral sugar (%)	Uronic acid (%)	Protein (%)
CC-1	31.95 ± 0.48	1.74 ± 0.03	24.83 ± 4.28
CC-2	52.49 ± 1.54	2.37 ± 0.48	21.69 ± 5.52

### 3.2 Molecular weight analysis

HPGPC analysis was used to determine the Mw of CC-2. A standard curve was established using dextran standards, yielding the equation: Y = −1.6395X + 36.973 (R^2^ = 0.9933), where X is the retention time and Y is Log Mw. The chromatogram of CC-2 (Figure 1) shows a single major, symmetrical peak, indicating a homogeneous polysaccharide fraction. The retention time of 20.251 min corresponded to an Mw of 5908.612 Da.

**FIGURE 1:**
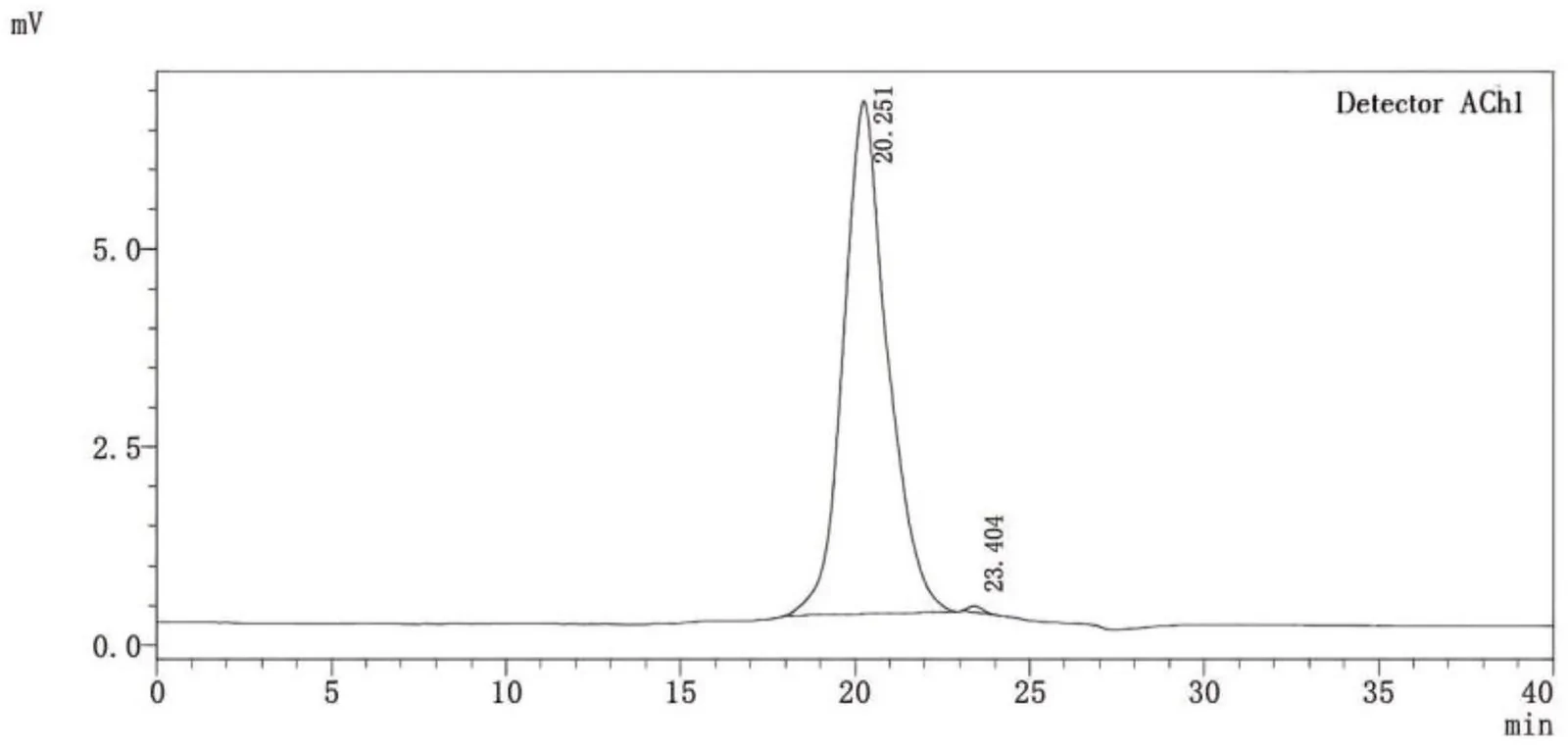
HPGPC chromatogram of the polysaccharide CC-2, showing its molecular weight distribution.

### 3.3 Monosaccharide composition

The monosaccharide composition of the polysaccharides is critical for their bioactivity. PMP-HPLC analysis (Figure 2) revealed that the crude polysaccharide CC-1 had a complex composition, predominantly glucose, with monosaccharides including rhamnose, glucuronic acid, galacturonic acid, glucose, galactose, xylose, and fucose in a molar ratio of 27:5:1:126:41:4:36. CC-2 showed a simpler profile, consisting mainly of rhamnose, glucuronic acid, glucose, galactose, and xylose in a molar ratio of 5:1:4:11:4.

**FIGURE 2:**
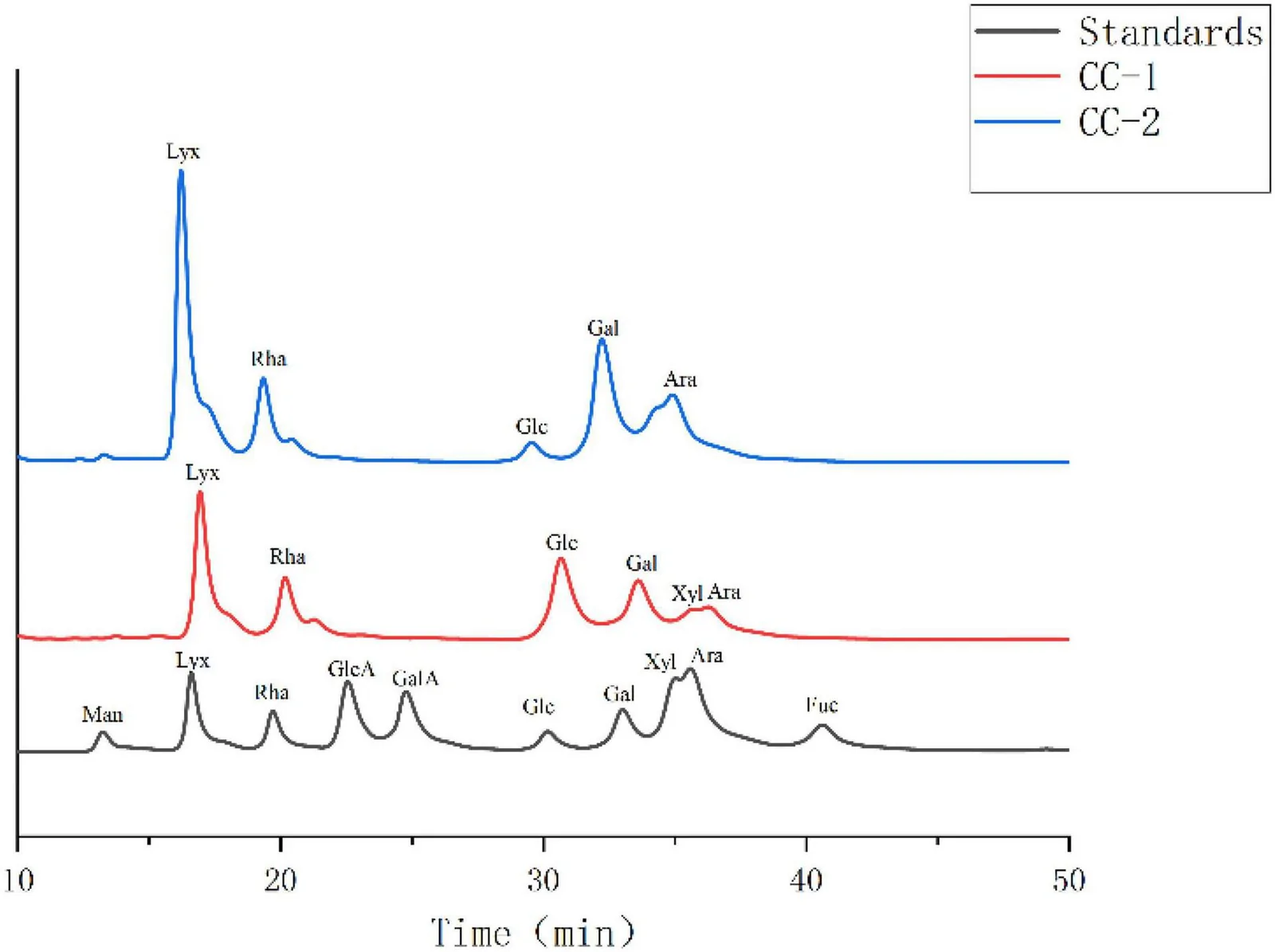
FT-IR spectra of CC-1 and CC-2.

### 3.4 UV, FT-IR, and particle size analysis

The UV spectrum of CC-2 (Figure 3) showed no significant absorption peak at 260 nm, confirming the absence of nucleic acids. The reduced absorption at 280 nm compared to CC-1 is consistent with a decrease in protein content, corroborating the chemical analysis.

**FIGURE 3:**
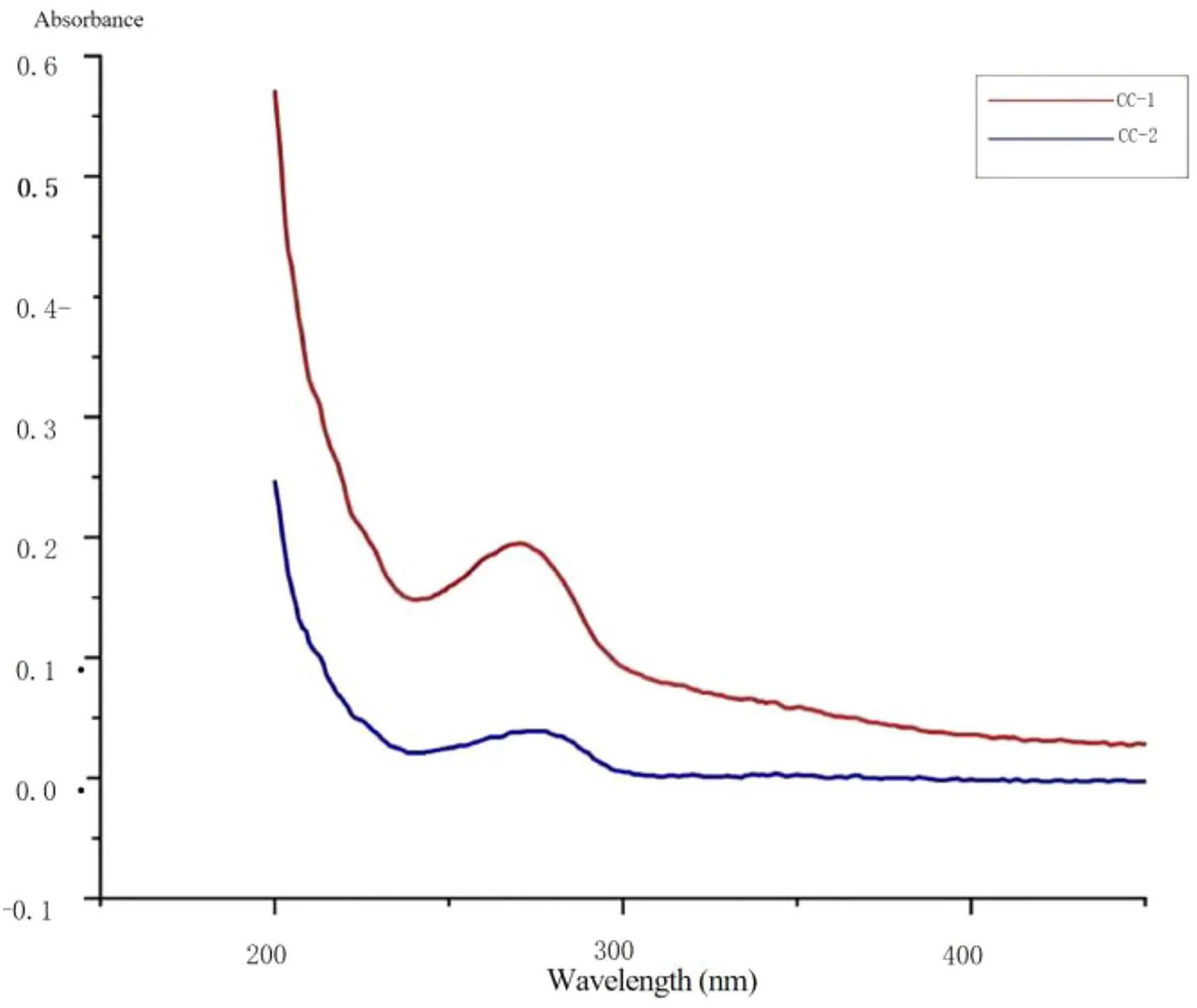
UV spectra of CC-1 and CC-2.

The FT-IR spectra of CC-1 and CC-2 (Figure 4) displayed typical polysaccharide absorption bands. A broad, intense peak around 3340 cm^−1^ corresponds to O–H stretching vibrations, and a peak near 2900 cm^−1^ is attributed to C–H stretching vibrations. A peak at 1644 cm^−1^ indicates the C=O stretching vibration of acetyl groups, suggesting that both polysaccharides contain acetyl groups. The absorption at 1074 cm^−1^, characteristic of a furanose ring, implies that the polysaccharides adopt a furan configuration.

**FIGURE 4:**
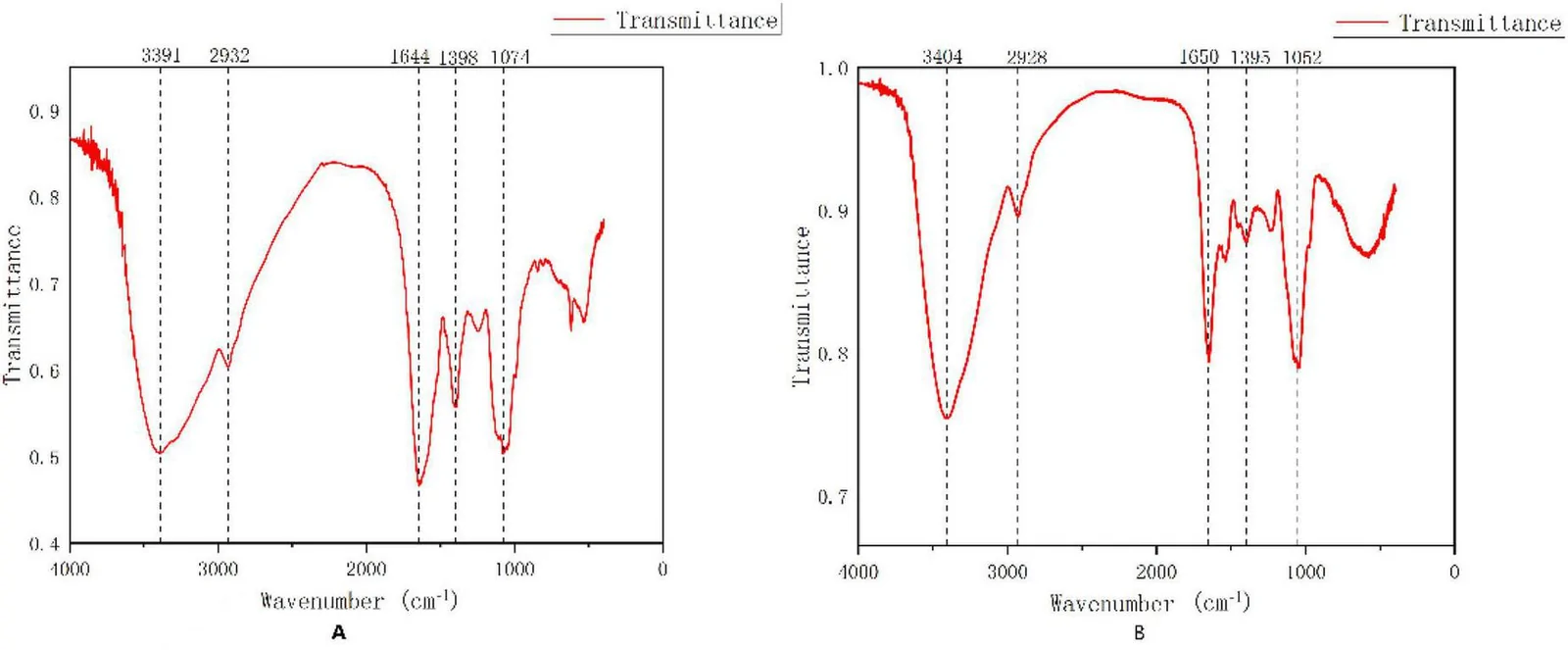
FT-IR spectra of **(A)** CC-1 and **(B)** CC-2.

Particle size analysis (Table 2) showed that the purification by dialysis significantly reduced the average particle size of the polysaccharide from 407.8 nm (CC-1) to 134.0 nm (CC-2) and decreased the polydispersity index (PdI) from 0.623 to 0.398. This suggests that dialysis effectively removed large aggregates, yielding smaller and more uniformly distributed polysaccharide particles in solution (Figure 5).

**TABLE 2:** Average particle size (Z-Ave) and polydispersity index (PdI) of polysaccharide samples (*n* = 3).

Sample	Z-Ave (nm)	PdI
CC-1	407.8 ± 35.1	0.623 ± 0.045
CC-2	134.0 ± 5.7	0.398 ± 0.057

**FIGURE 5:**
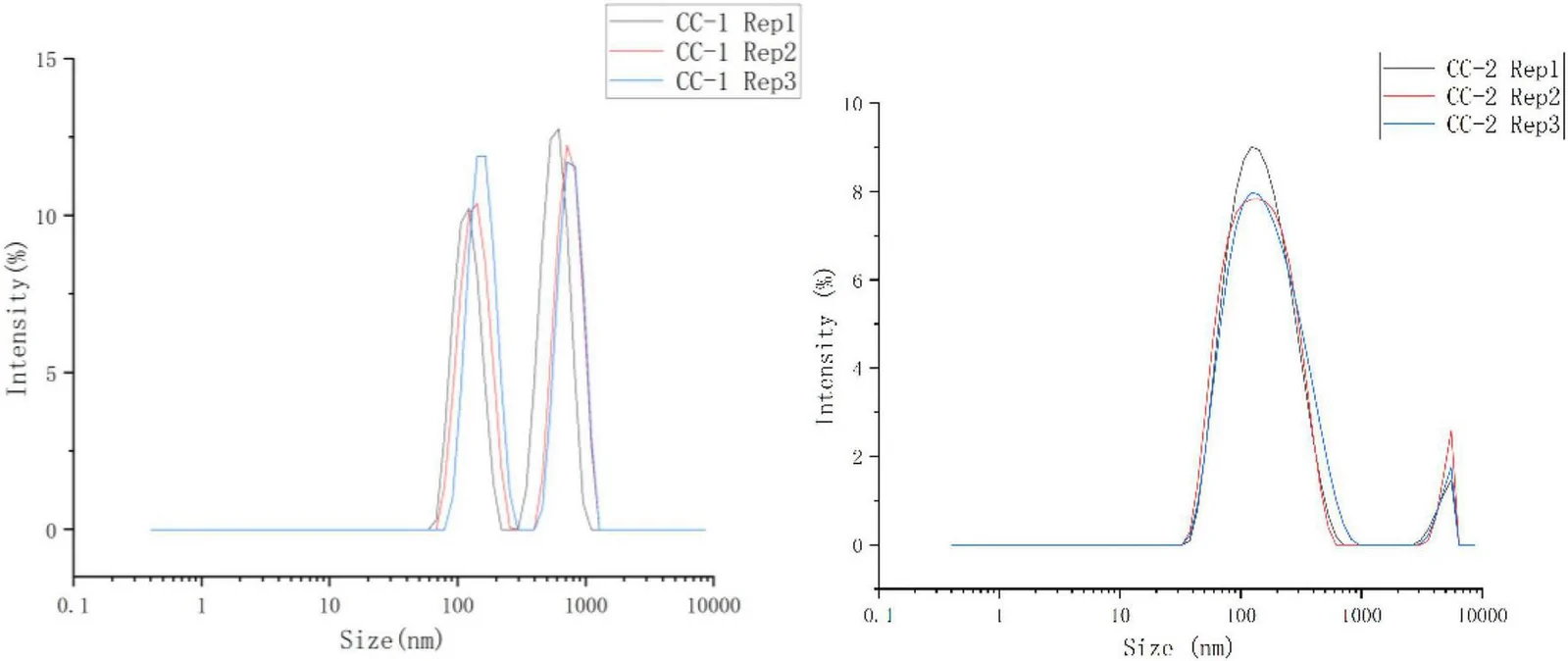
Particle size distribution profiles of CC-1 and CC-2 in aqueous solution.

### 3.5 Microstructural morphology

SEM was employed to observe the microstructure of the polysaccharide CC-2. As shown in Figure 6, the morphology is heterogeneous, consisting primarily of irregular, rough-surfaced blocks and numerous smooth spherical particles of varying sizes. This coexistence of two distinct morphologies may influence the physicochemical and biological properties of the polysaccharides, with the porous structure potentially providing a larger surface area for biological interactions.

**FIGURE 6:**
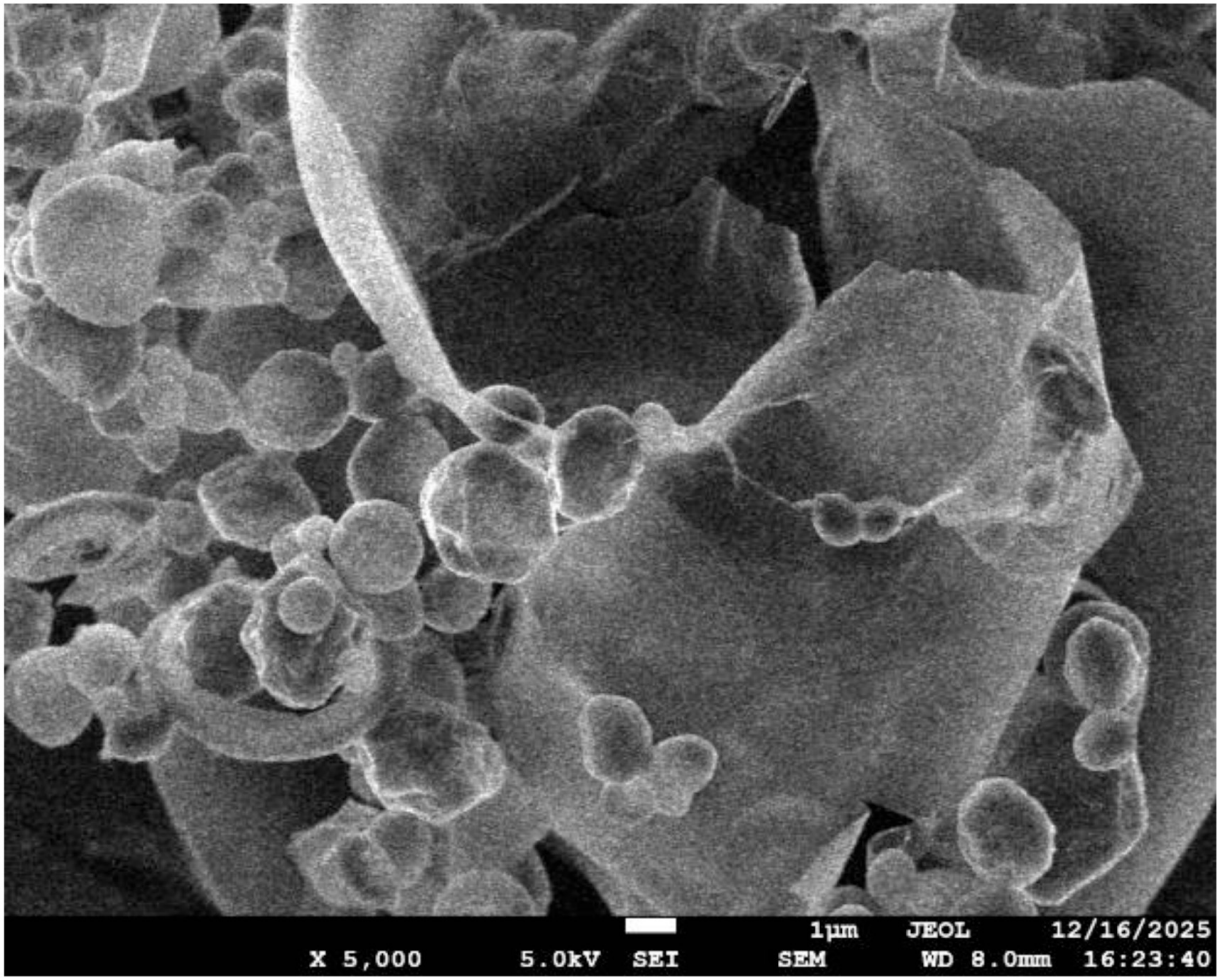
Scanning electron microscopy (SEM) images of *C. militaris* polysaccharide CC-2 showing its microstructure.

### 3.6 *In vitro* anticoagulant activity

The anticoagulant activity of CC-2 was assessed using the classic coagulation indicators APTT, PT, TT, and FIB. Breviscapine was used as the positive control, and saline as the blank. As summarized in Table 3, CC-2 at 5 mg/mL significantly prolonged PT, APTT, and TT compared to the blank control (*P* < 0.001), extending these times by approximately 2.10, 2.46, and 2.79 times, respectively. Furthermore, it significantly reduced the FIB concentration (*P* < 0.001). While the effect of CC-2 on prolonging PT, APTT, and TT was less potent than that of Breviscapine (20 mg/mL), its ability to lower FIB was significantly superior (*P* < 0.001). This finding is particularly noteworthy, suggesting that CC-2 may have a stronger effect on the final stage of the coagulation cascade or promote fibrinogen degradation.

**TABLE 3:** Effects of CC-2 on four coagulation parameters *in vitro* (*n* = 3).

Group	PT(s)	APTT(s)	TT(s)	FIB(g/L)
Blank (Saline)	6.73 ± 0.15	14.27 ± 0.25	15.47 ± 0.31	457.25 ± 0.63
Positive Control (Breviscapine, 20 mg/mL)	20.50 ± 0.26***	45.77 ± 0.31***	53.27 ± 0.35***	102.13 ± 0.74***
CC-2 (5 mg/mL)	14.17 ± 0.21***^#^	35.20 ± 0.36***^#^	43.20 ± 0.79***^#^	76.32 ± 0.90***^#^

***P < 0.001 vs. blank group;

^###^*P* < 0.001 vs. positive control group.

### 3.7 Discussion of anticoagulant mechanism and structure-activity relationship

Our results demonstrate, for the first time, the *in vitro* anticoagulant potential of the CC-2 from *C. militaris*. The observed prolongation of PT, APTT, and TT, together with the marked reduction in FIB, suggests that CC-2 may interfere with the coagulation cascade at multiple levels. Specifically, PT prolongation could reflect inhibition of the extrinsic pathway, while APTT prolongation might indicate disruption of the intrinsic pathway. The prolonged TT and decreased FIB further point to interference with the common pathway. However, it should be noted that inhibition restricted to the common pathway alone could produce a similar pattern of results. Therefore, the present data are consistent with, but do not definitively prove, a multi-pathway mechanism. The exact molecular targets of CC-2 within the coagulation cascade remain to be clarified through specific factor assays or selective inhibitor studies.

The biological activity of polysaccharides is closely related to their structural features. Mw is a key parameter. Our CC-2, with an Mw of ∼5.9 kDa, falls into the low-to-medium Mw range. Chen et al. (19) reported that sulfated polysaccharides with medium Mw (5–50 kDa) often exhibit better anticoagulant activity, as a suitable chain length is essential for binding to coagulation factors. An Mw that is too low might lack the necessary binding domains, while an Mw that is too high could lead to poor solubility and bioavailability. The potent activity of our low-Mw CC-2 could be due to enhanced exposure of active binding sites.

Monosaccharide composition is another critical factor. High galactose content has been positively correlated with anticoagulant activity, as galactose-containing polysaccharides can bind to thrombin (20). CC-2 is characterized by a high proportion of galactose (molar ratio 11 out of 25), which may contribute to its anticoagulant effect. However, Several structural aspects remain to be fully elucidated. Specifically, glycosidic linkage analysis have not been performed. Therefore, the current associations between high galactose content and the anticoagulant activity are correlative rather than mechanistic. Future work should focus on a more detailed structural analysis, including methylation – GC–MS, NMR spectroscopy and so on, to establish definitive structure–activity relationships.

## 4 Conclusion

This study successfully extracted, purified, and characterized polysaccharides from *Cordyceps militaris* (CC-2), revealing it to be a low-molecular-weight (5.9 kDa) heteropolysaccharide with a furan configuration, composed mainly of rhamnose, glucuronic acid, glucose, galactose, and xylose. Most importantly, CC-2 demonstrated significant *in vitro* anticoagulant activity by prolonging PT, APTT, and TT, and potently lowering FIB levels, with an efficacy superior to Breviscapine in reducing FIB. The prolongation of PT, APTT, and TT, along with the decrease in FIB, suggests that CC-2 may interfere with the coagulation cascade at multiple stages. These findings provide the first scientific evidence that *C. militaris* polysaccharides are a promising natural source of anticoagulant agents, with potential applications in functional foods and biomedicine for the prevention of thrombotic diseases. Future studies will focus on further elucidating the detailed structure, such as glycosidic linkage patterns, and validating these effects *in vivo* models.

## Data Availability

The original contributions presented in the study are included in the article/supplementary material, further inquiries can be directed to the corresponding authors.
